# Bone marrow mesenchymal stem cells as a possible ruxolitinib reservoir in the bone marrow niche

**DOI:** 10.1002/jha2.6

**Published:** 2020-05-12

**Authors:** Luigi Marino, Bruno Charlier, Valentina Giudice, Paolo Remondelli, Simona Paladino, Rosa Vitolo, Fabrizio Dal Piaz, Barbara Izzo, Pio Zeppa, Viviana Izzo, Amelia Filippelli, Carmine Selleri

**Affiliations:** ^1^ Department of Medicine Surgery and Dentistry “Scuola Medica Salernitana” University of Salerno Baronissi Italy; ^2^ Clinical Pharmacology University Hospital “San Giovanni di Dio e Ruggi d'Aragona” Salerno Italy; ^3^ Department of Molecular Medicine and Medical Biotechnology University of Naples “Federico II” Naples Italy; ^4^ Department of Molecular Medicine and Medical Biotechnology CEINGE‐Biotecnologie Avanzate University of Naples “Federico II” Naples Italy; ^5^ Pathology Unit University Hospital “San Giovanni di Dio e Ruggi d'Aragona” Salerno Italy; ^6^ Hematology and Transplant Center University Hospital “San Giovanni di Dio e Ruggi d'Aragona” Salerno Italy

1

Myelofibrosis (MF), a chronic myeloproliferative disorder, is characterized by early bone marrow (BM) hyperplasia and late‐stage fibrosis and splenomegaly. More than 50% of patients carry a somatic mutation (V617F) in the gene encoding for the Janus Kinase 2 (*JAK2*), a tyrosine kinase involved in several signaling pathways [1, 2], ultimately leading to uncontrolled proliferation and cell survival [3]. In 2011, a JAK1/2 inhibitor, ruxolitinib, has been approved for treatment of primary MF (PMF) [4, 5]. This drug competitively inhibits the JAK2 ATP‐binding catalytic site and decreases STAT5 activity, downregulates inflammatory signaling pathways, and reduces hematopoietic stem cell (HSC) proliferation rate especially in those cells harboring the *JAK2*V617F mutation [3]. BM‐mesenchymal stem cells (MSCs) could modify the BM niche composition, ameliorate fibrosis during various treatments [6], and might also modulate uptake, release, and metabolism of ruxolitinib. The aim of this study was to investigate in vitro the uptake rate of ruxolitinib by bone marrow mesenchymal stem cells (BM‐MSCs) derived from MF patients and to study the effects of treated BM‐MSCs and conditioned culture media on leukemic cell growth in order to add further information to ruxolitinib mechanism of action in MF.

BM heparinized specimens were collected from five patients with PMF or postpolycythemia vera myelofibrosis (PV‐MF) carrying the *JAK2*V617F mutation (Figure 1A) after informed consent obtained in accordance with protocols approved by the Ethic Committee of Our Institution (ASL Napoli 3 Sud, Naples, Italy; prot./SCCE n. 24988). Patients received a diagnosis of PMF or PV‐MF according to the 2016 WHO criteria (BM histology of two representative patients in Figure 1B). A total of 5 × 10^4^ BM mononuclear cells/cm^2^ was seeded in the presence of alpha‐MEM with l‐glutamine supplemented with 10% FBS (Corning Inc., Corning, NY) and antibiotics (penicillin 100 U/mL, streptomycin 100 mg/mL, and amphotericin B 0.25 mg/mL; Biowest, Nuaillé, France). MSC colony growth was monitored for 14 days; then, colonies were detached by trypsinization (4.5 mL/Flask of 0.025% Tripsin/EDTA, for 7 min at +37°C), and cells were seeded at 4000 cells/cm^2^. BM‐MSCs were expanded up to the third passage and mesenchymal phenotype was confirmed according to the International Society of Cellular Therapy guidelines [7]. A total of 1 × 10^5^ cells was stained with the following antibodies: CD90‐FITC or HLA‐DR‐FITC; CD105‐PE or CD3‐PE; CD73‐APC; and CD45‐PE‐Cy7 or CD14‐PE‐Cy7 (Beckman Coulter, Milan, Italy). Samples were acquired using a BD FACSVerse flow cytometer. All primary cell lines displayed: (i) the ability to adhere to tissue culture plastics; (ii) fibroblast‐like spindle shape (Figure 1C left); (iii) positivity for mesenchymal markers (CD90, CD105, and CD73); and (iv) negativity for CD34, CD14, CD45, and HLA‐DR (Figure 1C right). The in vitro differentiation ability toward osteogenic, adipogenic, and chondrogenic cells was not tested because of the small number of BM‐MSCs obtained at the third passage. After established BM‐MSC primary cell lines (named MPN‐1 to ‐5), cells were stored in 10% dimethyl sulfoxide (DMSO) and 50% human serum albumin at −80°C until use.

**FIGURE 1:**
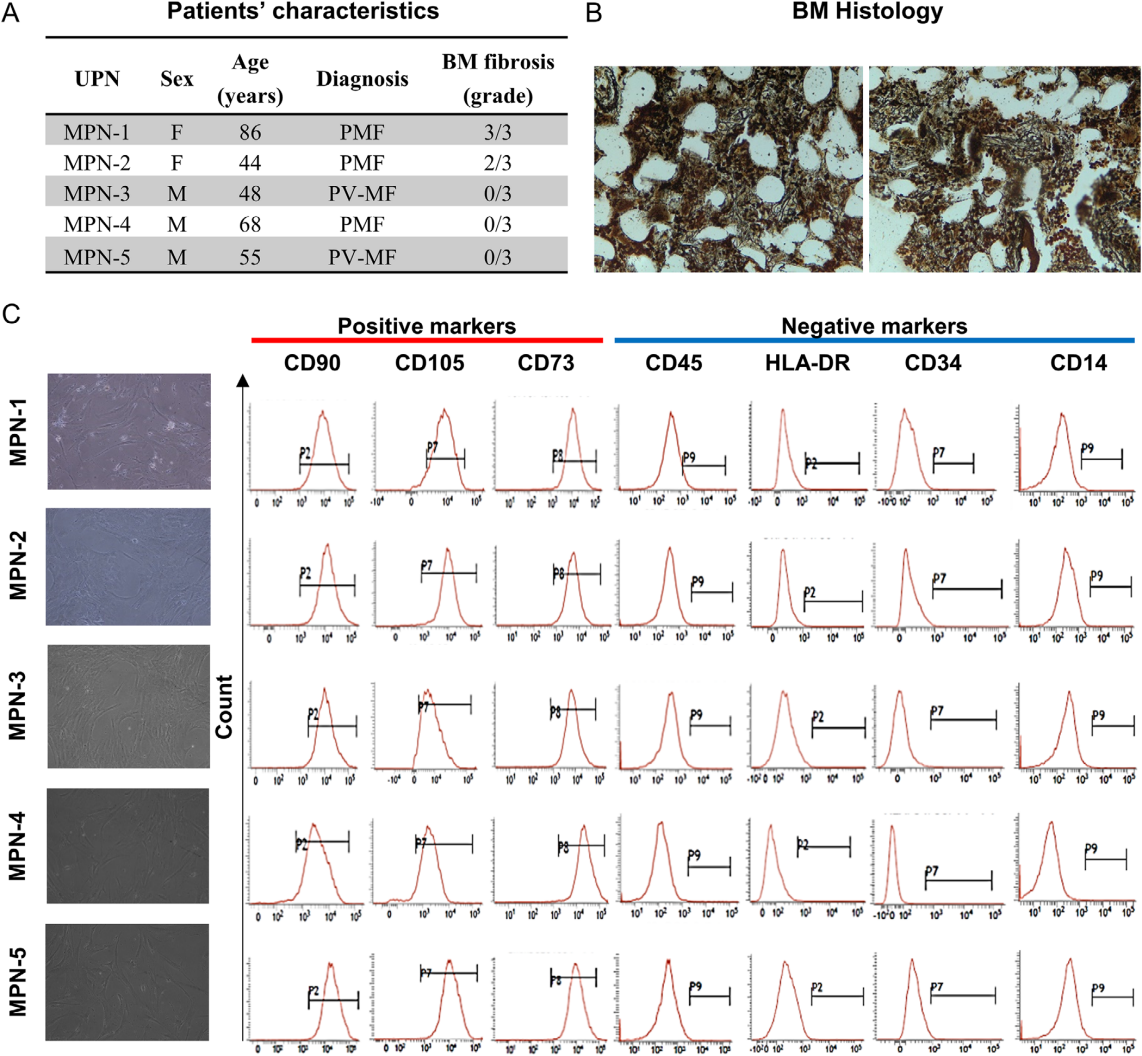
Patients’ and bone marrow mesenchymal stem cell (BM‐MSC) primary cell lines’ characteristics. A, Clinical characteristics of enrolled patients diagnosed with primary myelofibrosis (PMF) or postpolycythemia vera myelofibrosis (PV‐MF) according to the 2016 WHO criteria. B, Two representative BM histology samples of various grades of fibrosis documented by a reticulin immunohistochemistry staining. C, After establishment of BM‐MSC cell line, the mesenchymal phenotype was confirmed for each cell line (MPN‐1 to ‐5) following the International Society of Cellular Therapy guidelines. (C, left) We observed the fibroblast‐like spindle shape using an INV‐100T phase contrast microscope (Eurotek, Eatontown, NJ) (10X magnification). (C, right) Flow cytometry analysis showed the positivity of cell lines for CD90, CD105, and CD73, and the negativity for other lineage specific surface markers, such as CD45, HLA‐DR, CD34, and CD14. Data are shown as cell count histograms for each antibody used and gates defined on total live cells from isotype controls and unstained specimens used as negative controls. Data were analyzed using the BD FACSuite software

To evaluate the ability of BM‐MSCs to incorporate ruxolitinib, (provided by Novartis) *JAK2*+ BM‐MSCs were treated at various drug concentrations (0.5, 2.5, and 5 μg/mL) for 48 h, and ruxolitinib uptake was documented by confocal microscopy using a LSM 700 microscope (Carl Zeiss Microimaging, Inc. Jena, Germany) equipped with planapo 63 × oil‐immersion [NA 1.4] objective lens. A 405 nm diode laser for excitation and a 450 nm band pass filter for emission were used because ruxolitinib can be detected by fluorimetry with excitation and emission wavelengths of 320 and 386 nm, respectively [8]. Images were acquired with the confocal pinhole set to one Airy unit (AU) using the same setting (laser power, detector gain) for all experimental conditions. BM‐MSCs in ruxolitinib‐free complete medium were used as negative control; while, HeLa cells, a cervical cancer cell line known to have JAK/STAT pathway hyperactivation [9], was employed as positive control. A dose‐dependent uptake of ruxolitinib by BM‐MSCs was observed (Figure 2A) with a mean fluorescence intensity of 800 ± 50 AU at 1 μg/mL and 1300 ± 90 AU at 2.5 μg/mL with a plateau at 5 μg/mL (1500 ± 110 AU) and increased cell death. Similarly, HeLa cells showed a dose‐dependent drug uptake and changes in morphology at higher concentrations (Figure 2B). In addition, BM‐MSCs displayed a spot‐crystal like pattern of drug uptake. MSCs can be easily isolated and engineered to produce anticancer drugs and immunomodulatory cytokines [10, 11]. After reinjection, MSCs can migrate to the site of inflammation; however, cells poorly traffic from periphery to target sites because of the lack of specific tissue‐homing signals [10]. Therefore, engineered MSCs mainly act at distance realizing drug‐loaded exosomes or by polarizing macrophages/monocytes [10, 12]. Moreover, MSCs can incorporate drugs and release active biomolecules directly into tumor microenvironment [12, 13]. Our results showed that BM‐MSCs could uptake ruxolitinib when exposed in vitro to high drug concentrations and act as a reservoir. However, further studies are required to elucidate if ruxolitinib uptake is dependent of cell type and/or presence or absence of *JAK2* mutations.

**FIGURE 2:**
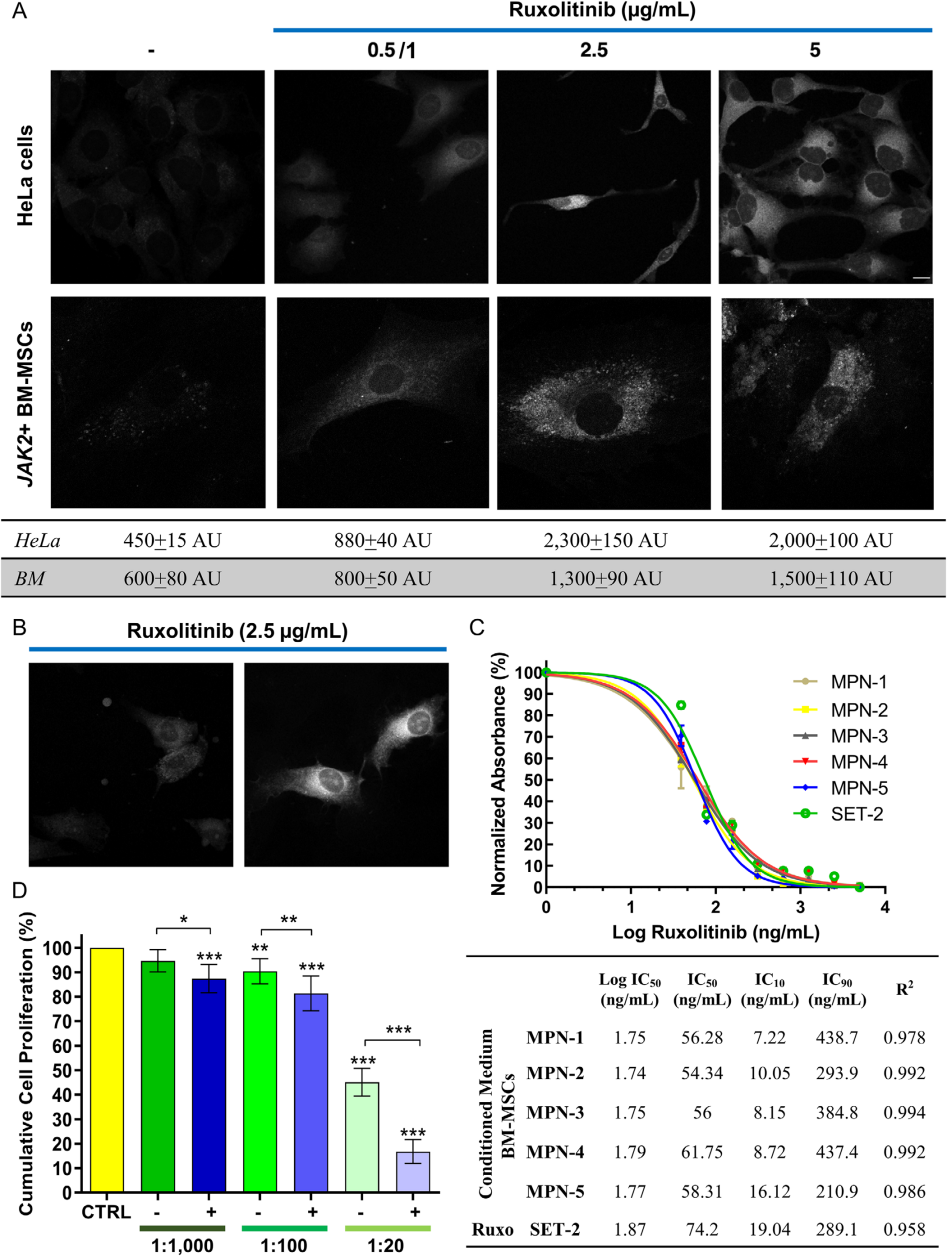
Ruxolitinib uptake by BM‐MSCs and antiproliferative effects on leukemic stem cells. A, Ruxolitinib was added at various concentrations (0, 0.5 or 1, 2.5, and 5 μg/mL) to HeLa cells or BM‐MSCs and incubated for 48 h. Then, fluorescence intensity was measured by confocal microscopy (LSM 700, Carl Zeiss Microimaging, Inc., Thornwood, NY) at 405/490 with background subtraction at 490 nm in a dark region of the field. A dose‐dependent uptake of ruxolitinib was documented with a plateau at 5 μg/mL. However, only few BM‐MSCs were observed at 5 μg/mL and fluorescence could not be representative at this concentration. B, Changes in morphology with spindle shape cells were described at higher drug concentrations in HeLa cells. C, inhibitory concentrations (ICs) 10, 50, and 90 were determined using the SET‐2 cell line as a model of leukemic cell line carrying the *JAK2*V617F mutation. Serial dilutions of ruxolitinib (Ruxo; 5 μg/mL to 1 ng/mL) or conditioned medium (CM) obtained from each of the five BM‐MSC primary cell lines (MPN‐1 to ‐5) were prepared, and the proliferation rate was assessed by colorimetric CCK‐8 assay after a 7‐day incubation. Absorbance was read at 450 nm for each sample in duplicate, and values were used to calculate a dose‐response inhibition curve normalized on the mean OD_450nm_ value from untreated SET‐2 cells. The *R* ^2^ values are also reported. (D) Each BM‐MSC primary cell line was treated or not (− or +) with ruxolitinib at 5 μg/mL and cocultured for 7 days with SET‐2 cells at different seeding ratios: 1:20; 1:100; and 1:1000. Cumulative cell proliferation rates were assessed by CCK‐8 assay on each sample in duplicate, and data normalized using the absorbance obtained from SET‐2 cells (CTRL). Data are shown as mean ± *SD*. To evaluate group differences, one‐way ANOVA with Tukey's multiple comparison test was performed and a *P* < .05 considered statistically significant. *P < .05; ^**^ *P* < .01; ^***^ *P* < .001

Next, the antiproliferative effects on leukemic cells of conditioned medium (CM) obtained from BM‐MSCs treated with 5 μg/mL of ruxolitinib for 24 h were explored. First, inhibitory concentrations (ICs) 10, 50, and 90 were determined using the SET‐2 cell line (ACC‐608; DSMZ) as a model of leukemic cell line carrying the *JAK2*V617F mutation. Serial dilutions of ruxolitinib or CM (starting concentration, 5 μg/mL) obtained from each of the five BM‐MSC primary cell lines were prepared, and 2 × 10^4^ SET‐2 cells were added for a final volume of 100 μL/well. After a 7‐day incubation, proliferation rate was assessed by CCK‐8 assay (Sigma–Aldrich, St. Louis, MO) according to manufacturer's instructions. Absorbance was read for each sample in duplicate using a Tecan I‐control Infinite 200 PRO plate reader, and values were used to calculate a dose‐response inhibition curve (Figure 2C). Mean OD_450nm_ value from untreated SET‐2 cells (mean OD_450nm_ = 3.696) was used for data normalization; while, a blank sample containing culture medium was used for background subtraction. The IC50 of CM from primary ruxolitinib‐treated BM‐MSCs was significantly lower than that of ruxolitinib alone (mean IC50, 57.3 ng/mL vs 74.2 ng/mL, respectively; *P* = .006 by unpaired *t*‐test). The IC10 of CM from treated BM‐MSCs was slightly lower than ruxolitinib alone (10.1 ng/mL vs 19 ng/mL, respectively; *P* = .082 by unpaired *t*‐test), because the MPN‐5 cell line had an IC10 similar to that from control. No differences were found between the IC90 of CM and ruxolitinib (mean IC90, 353.1 ng/mL vs 289.1 ng/mL, respectively; *P* = .586; unpaired *t*‐test performed). Our data suggested that CM obtained from BM‐MSCs of patients with MF treated with ruxolitinib could have an antiproliferative effect on leukemic cell growth higher than that of ruxolitinib alone. These preliminary findings need further investigation in order to clarify if the increased antiproliferative action might be caused by drug‐loaded exosomes released from BM‐MSCs, as described for Paclitaxel‐exposed MSCs [12], or by cytokine release in response to ruxolitinib.

Finally, we explored the in vitro antiproliferative effects on SET‐2 cells. Each BM‐MSC primary cell line was treated or not with ruxolitinib at 5 μg/mL and cocultured for 7 days with SET‐2 cells at different seeding ratios (BM‐MSC:SET‐2): 1:20; 1:100; and 1:1000. Proliferative rates were assessed by CCK‐8 assay on each sample in duplicate. Data were normalized using values obtained from SET‐2 cells grown without ruxolitinib (OD_450nm_ = 3.679). Proliferation decreased in both treated and untreated samples by increasing the number of BM‐MSCs in cocultures. However, SET‐2 cells cocultured with ruxolitinib‐treated BM‐MSCs showed a higher reduction in the proliferation rates compared to cultures with untreated BM‐MSCs for all seeding ratios (Figure 2D). In particular, for 1:20 ratio, the cumulative cell proliferation rate was 16.8% in ruxolitinib‐treated cultures compared to 45.1% in untreated samples (*P* < .0001; unpaired *t*‐test performed). For 1:100 and 1:1000 ratios, rates were 81.4% vs 90.4% or 87.4% vs 94.7%, ruxolitinib‐treated vs untreated samples, respectively (*P* = .004 or *P* = .006; unpaired *t*‐test performed). Our preliminary data showed a possible antiproliferative effect of ruxolitinib‐treated BM‐MSCs on leukemic stem cells that increased by augmenting the number of MSCs in culture.

The present work is an exploratory study to evaluate ruxolitinib uptake by BM‐MSCs and to investigate the antiproliferative effects of ruxolitinib‐treated mesenchymal cells on leukemic cell growth; however, several limitations are present. First, we performed our exploratory work using only BM‐MSCs from patients with MF carrying the *JAK2*V617F mutation; therefore, further studies using BM‐MSCs not harboring the mutation or other cell types, such as HSCs, need to be performed. Second, our results raised the hypothesis that ruxolitinib‐treated BM‐MSCs might alter culture medium composition; thus, additional investigations for determining cytokine levels and exosome composition need to be performed.

Our results suggested that ruxolitinib‐treated BM‐MSCs might influence leukemic cell growth by direct cell‐to‐cell interaction or by indirect release of drug‐loaded exosomes and cytokines in the medium/microenvironment [13, 14, 15]. Ruxolitinib‐treated BM‐MSCs might act not only as a passive drug reservoir but also as disease modifiers, making those cells a good candidate for MF cellular therapy. However, further studies need to be performed in order to shed lights on additional mechanisms of action of ruxolitinib in the treatment of bone marrow fibrosis and myeloproliferative disorders.

## CONFLICT OF INTEREST

The authors declare that there is no conflict of interest that could be perceived as prejudicing the impartiality of the research reported.

## AUTHOR CONTRIBUTIONS

L.M., V.G., V.I., and C.S. participated in the design of the study. L.M, B.C., V.G., S.P., R.V., F.D.P., and B.I. conducted the experiments and analyzed the data. L.M., V.G., P.R., A.F., P.Z., and C.S. interpreted the results and drafted the manuscript. P.R., S.P., F.D.P., B.I., V.I., P.Z., and A.F. edited the manuscript. All authors critically reviewed the manuscript content and agreed with the final submission of the manuscript.
